# 

**Published:** 1913-09

**Authors:** 


### CONTENTS.

### ORIGINAL COMMUNICATIONS—

End Results in Gall Bladder Surgery. By Dr. E. L. Griffin, Atlanta, Ga. .......... 237
The Practical Aid of the Wassermann Reaction to the General Practitioner. By C. W. Gould, M. D., Atlanta, Ga. .......... 240
Medical and Dental Examination of Children in the Public Schools. By J. L. Bowman, M. D., Union Springs, Ala. .......... 243
Why and When Bacterial Vaccines Should be Given. By W. Frank Wells, M. D., Atlanta, Ga. .......... 250
A Short Study of Alcohol and its Effects on the Human Body. By W. C. Ashworth, M. D., Greensboro, N. C. .......... 254
Vulvo-Vaginitis in Infants and Children. By W. N. Adkins, M. D., Atlanta, Ga. .......... 267
EDITORIAL .......... 273
SELECTIONS AND ABSTRACTS .......... 277
BOOK REVIEWS .......... 287

